# Diagnostic Utility of Fasting Blood Glucose and Glycated Hemoglobin Compared to Oral Glucose Tolerance Test for Diabetes Mellitus: A Cross‐Sectional Study

**DOI:** 10.1002/hsr2.73507

**Published:** 2026-11-10

**Authors:** Emmanuel Okrah, Dawudu Abdullah, Beatrice Manu, James Kojo Prah, Samuel Amoah, Benjamin Nyane, Gloria N. Nartey, Eugenia N. A. Ayetey, Ibrahim Anyass Goumboundi, Justice Afrifa, Richard K. D. Ephraim

**Affiliations:** ^1^ Department of Medical Laboratory Science University of Cape Coast Cape Coast Ghana

**Keywords:** diabetes mellitus, HbA1c, oral glucose tolerance test (OGTT), screening

## Abstract

**Introduction:**

Fasting blood glucose (FBG), glycated hemoglobin (HbA1c), and the oral glucose tolerance test (OGTT) may classify individuals differently when used to assess diabetes mellitus. This exploratory study compared diabetes classification by these methods and evaluated the agreement and diagnostic performance of HbA1c and FBG relative to the study's 2‐h post‐glucose‐load criterion among adults at increased risk of diabetes attending the University of Cape Coast Hospital, Ghana.

**Method:**

A hospital‐based cross‐sectional study was conducted among 50 adults with one or more diabetes mellitus risk factors attending the University of Cape Coast Hospital, Ghana. Participants underwent FBG, HbA1c, and a 75‐g OGTT on the same day. Diabetes mellitus was defined as FBG ≥ 7.0 mmol/L, 2‐h post‐glucose‐load glucose ≥ 11.1 mmol/L, or HbA1c ≥ 6.5%, according to international diagnostic criteria. Diagnostic agreement between HbA1c and the 2‐h post‐glucose‐load classification was assessed using Cohen's kappa statistic, while sensitivity, specificity, positive predictive value (PPV), and negative predictive value (NPV) of HbA1c and FBG were calculated using the 2‐h post‐glucose‐load classification as the reference standard.

**Results:**

Among 50 participants, seven (14.0%) met the study's 2‐h post‐glucose‐load criterion for diabetes mellitus, two (4.0%) met the HbA1c criterion, and none met the FBG criterion. Agreement between HbA1c and the post‐load classification was 86.0% (Cohen's *κ* = 0.17). Relative to the post‐load criterion, HbA1c sensitivity was 14.3% (95% CI: 0.4%–57.9%) and specificity was 97.7% (95% CI: 87.7%–99.9%). FBG sensitivity was 0.0% (95% CI: 0.0%–41.0%), and specificity was 100.0% (95% CI: 91.8%–100.0%).

**Conclusion:**

The 2‐h post‐glucose‐load criterion identified more participants with diabetes‐range results than HbA1c or FBG in this sample. Although HbA1c demonstrated excellent specificity, its low sensitivity suggests that it may miss a considerable proportion of diabetes cases when used as a standalone diagnostic test. These findings support the continued use of OGTT as the reference method for diagnosing diabetes mellitus in high‐risk individuals, particularly where early detection is critical. However, larger, well‐powered multicentre studies are needed to validate these results.

## 1 Introduction

Diabetes mellitus is a chronic metabolic disorder characterized by persistent hyperglycemia resulting from inadequate insulin production, ineffective insulin action, or both [1]. The global prevalence of diabetes mellitus is rising rapidly, with estimates indicating 9.3% (463 million people) in 2019, projected to increase to 10.2% (578 million) by 2030 and 10.9% (700 million) by 2045 [2]. The burden is particularly heavy in developing countries, which are expected to see a 69% increase in diabetes mellitus cases between 2010 and 2030, compared to a 20% increase in developed countries [3]. These findings underscore the urgent need for early detection, effective prevention, and management strategies worldwide. Early and accurate diagnosis of diabetes mellitus is essential for managing the disease and reducing its complications; however, current diagnostic methods vary in their effectiveness and predictive value. The diagnosis of diabetes mellitus has traditionally been based on blood glucose levels, assessed through tests like the Oral Glucose Tolerance Test (OGTT) and Fasting Blood Glucose (FBG). While these methods are effective, they require fasting and/or postprandial sampling, which can be inconvenient and may not be feasible in all settings [4].

In recent years, glycated hemoglobin (HbA1c) has gained acceptance as a diagnostic tool due to its ability to reflect average blood glucose levels over several months, eliminating the need for fasting. HbA1c is associated with less intra‐individual variability and is more reproducible than the FBG and 2‐h post‐glucose‐load glucose measurements. Despite their widespread use, each diagnostic test has important limitations. FBG and OGTT require fasting, while OGTT is time‐consuming and less convenient in routine clinical practice. Conversely, HbA1c reflects long‐term glycaemic exposure and does not require fasting; however, its diagnostic performance may be influenced by conditions independent of glycemia, including anemia, hemoglobinopathies, chronic kidney disease, and altered erythrocyte turnover [5]. These conditions are particularly relevant in sub‐Saharan Africa, where hemoglobin disorders are relatively common.

Although FBG, HbA1c, and OGTT are established diagnostic tests, evidence comparing their performance among Ghanaian adults at increased risk of diabetes remains limited. Understanding the degree of agreement between these tests and their ability to identify diabetes within this population is important for informing appropriate diagnostic strategies in resource‐limited settings.

Studies from sub‐Saharan Africa have demonstrated differences in diabetes mellitus classification using HbA1c and glucose‐based criteria. In the Durban Diabetes Study, HbA1c ≥ 6.5% had a sensitivity of 70.3% and specificity of 98.7% relative to OGTT among participants without previously diagnosed diabetes [6]. In Tanzania, Jeremiah et al. [7] studied adults with and without HIV and reported diabetes prevalences of 13% using HbA1c and 6% using OGTT, with only partial overlap between the participants identified by the two methods. These findings illustrate that the direction and extent of disagreement can differ across populations and settings.

Locally relevant comparisons are therefore needed to understand how these tests classify adults undergoing glucose assessment in Ghana. The present exploratory study compared the proportions meeting diabetes criteria using FBG, HbA1c and 2‐h post‐glucose‐load glucose, assessed agreement between HbA1c and the post‐load classification, and estimated the diagnostic performance of HbA1c and FBG relative to the study's post‐load criterion among adults at increased risk of diabetes attending the University of Cape Coast Hospital.

## 2 Methods

### 2.1 Study Design and Setting

This hospital‐based cross‐sectional study was conducted at the University of Cape Coast Hospital, Cape Coast, Ghana, between November 2024 and August 2025. The University of Cape Coast Hospital serves the university community and surrounding populations by providing primary and secondary healthcare services, including routine diabetes screening and diagnostic testing. A cross‐sectional design was considered appropriate because it enabled the simultaneous comparison of FBG, HbA1c, and 2‐h post‐glucose‐load glucose results among adults at increased risk of diabetes mellitus. Purposive sampling was employed to recruit individuals with established diabetes mellitus risk factors in order to ensure adequate representation of the target population for diagnostic comparison. Although this sampling strategy limits the generalisability of the findings, it was considered appropriate for this exploratory diagnostic evaluation.

### 2.2 Study Population

The study included adults aged 18 years and above attending the University of Cape Coast Hospital for routine glucose assessment who had at least one recognised risk factor for type 2 diabetes mellitus. Risk factors included obesity (body mass index ≥ 30 kg/m^2^), hypertension, a first‐degree family history of diabetes mellitus, previous gestational diabetes mellitus, physical inactivity, or age ≥ 45 years, consistent with the American Diabetes Association recommendations.

### 2.3 Sample Size Determination

This study was designed as an exploratory cross‐sectional diagnostic comparison to generate preliminary evidence on the performance of FBG and HbA1c relative to the 2‐h post‐glucose‐load reference classification among adults at increased risk of diabetes. A purposive sample of 50 eligible participants was recruited. Although a formal sample size calculation was not performed, the relatively small sample size is acknowledged as a limitation, as it may have reduced statistical power, limited the precision of the diagnostic performance estimates, and restricted the generalisability of the findings.

### 2.4 Eligibility Criteria

Adults aged 18 years or older with at least one recognised diabetes mellitus risk factor who consented to participate were eligible for inclusion. Individuals with a previous diagnosis of diabetes mellitus or receiving glucose‐lowering therapy were excluded. Participants with conditions known to influence glucose metabolism or HbA1c measurements, including pregnancy, chronic kidney disease, chronic liver disease, hemoglobinopathies, anemia, recent blood transfusion, or corticosteroid therapy, were also excluded.

### 2.5 Data Collection Procedure

A structured interviewer‐administered questionnaire was used to obtain demographic characteristics (age, sex, marital status and educational level), behavioral factors (physical activity, smoking, alcohol consumption and dietary habits), family history of diabetes mellitus and symptoms suggestive of hyperglycemia. Anthropometric measurements were obtained by trained research personnel using standardised procedures.

### 2.6 Anthropometric Measurements

Anthropometric measurements, including participants' weight (kg) and height (cm) was measured with a weight and height scale‐FULLMEDI (FM‐S120, China). Body weight was measured to the nearest 0.1 kg using a calibrated digital weighing scale with participants wearing light clothing and no shoes. Height was measured to the nearest 0.1 cm using a portable stadiometer. Body mass index (BMI) was calculated as weight (kg) divided by height squared (m^2^). BMI was classified according to the World Health Organization criteria as underweight (< 18.5 kg/m^2^), normal weight (18.5–24.9 kg/m^2^), overweight (25.0–29.9 kg/m^2^), and obesity (≥ 30.0 kg/m^2^) (World [8]).

### 2.7 FBG Estimation

Participants were instructed to fast overnight for 8–12 h before blood collection. Venous blood samples were collected into fluoride oxalate tubes by trained phlebotomists. Plasma glucose concentration was measured using an enzymatic glucose oxidase method according to the manufacturer's instructions. Diabetes mellitus was defined as a fasting plasma glucose concentration ≥ 7.0 mmol/L in accordance with the American Diabetes Association diagnostic criteria [9]. Internal quality control materials were analysed daily before patient samples to ensure analytical accuracy.

### 2.8 OGTT Estimation

Participants fasted overnight for 8–12 h before the OGTT. Following skin disinfection and air drying, venous blood was collected, and fasting plasma glucose was measured using an enzymatic method. Participants then consumed a solution containing 75 g anhydrous glucose dissolved in 250 mL of water over 5 min [10].

Capillary blood glucose was measured at 1 and 2 h after glucose administration using the OneTouch Select Plus glucometer (LifeScan Inc., USA); a handheld glucometer. Before each measurement, the fingertip was cleaned with an alcohol swab and allowed to air dry. A sterile lancet was used to puncture the skin, the first drop of blood was discarded, and the second drop was applied to a test strip for measurement. Results were recorded in mmol/L. The 2‐h post‐glucose‐load measurement was used for the study's reference classification. The thresholds applied were ≥ 11.1 mmol/L for diabetes and 7.8–11.0 mmol/L for impaired glucose tolerance.

### 2.9 HbA1c Estimation

Regard to HbA1c, no special preparation like fasting is needed. Blood was taken from the median cubital vein in the antecubital fossa and were processed using a Quo‐Lab™ HbA1c Analyzer following manufacturer's protocol for HbA1c estimation. The analyser uses boronate affinity methodology, and results were expressed as a percentage of total hemoglobin that is glycated, reflecting average blood sugar levels over the past 2 to 3 months. Results were recorded using the current diagnostic criteria for HbA1c include: normal (< 5.7% or < 39 mmol/mol), prediabetes (5.7%–6.4% or 39‐47 mmol/mol), and diabetes (≥ 6.5% or ≥ 48 mmol/mol) by Mathew et al. [11].

### 2.10 Ethical Considerations

Ethical approval for this study was obtained from the Institutional Review Board of the University of Cape Coast Hospital (Reference No. UCC‐184/2025). Written informed consent was obtained from all participants before enrollment. All procedures complied with the ethical principles of the Declaration of Helsinki. Participant confidentiality was maintained throughout the study by anonymising all collected data.

### 2.11 Data Analysis

Data were entered into Microsoft Excel and analysed using IBM SPSS Statistics version 29.0 (IBM Corp., Armonk, NY, USA). Continuous variables were assessed for normality using the Shapiro–Wilk test. Normally distributed variables are presented as mean ± standard deviation (SD), whereas non‐normally distributed variables are summarised as median (interquartile range). Categorical variables are presented as frequencies and percentages. Comparisons between groups were performed using the independent samples *t*‐test or Mann–Whitney *U* test for continuous variables, as appropriate, and the Chi‐square test or Fisher's exact test for categorical variables. Agreement between HbA1c and the 2‐h post‐glucose‐load classification was evaluated using Cohen's kappa statistic and interpreted according to the criteria proposed by Landis and Koch. Diagnostic performance measures, including sensitivity, specificity, positive predictive value (PPV), and negative predictive value (NPV), were calculated using the 2‐h post‐glucose‐load classification as the reference standard. Where appropriate, 95% confidence intervals were calculated. All statistical tests were two‐sided, and a *p* < 0.05 was considered statistically significant.

## 3 Results

Table 1 summarizes the general characteristics of the 50 study participants. Females constituted 56% and males 44%, with mean ages of 46.46 ± 15.59 and 44.73 ± 16.65 years, respectively. Most participants were aged ≥ 40 years (71.4% of females; 63.6% of males). University graduates formed the largest group (46.4% females; 54.5% males), while primary school graduates were the fewest. The majority were married (60.7% females; 59.1% males). Among females, 42.9% were overweight and 39.3% obese; among males, 31.8% were overweight and 4.5% obese. No significant sex differences were found for age, marital status, or weight (*p* > 0.05). However, BMI (*p* = 0.002) and height (*p* < 0.001) differed significantly between males and females. Females were more likely to be overweight or obese than males. These differences should be considered when interpreting the study findings, although no stratified analyses were performed because of the limited sample size.

**Table 1:** General characteristics of the study population.

Variable	Female (*N* = 28)	Male (*N* = 22)	Total (*N* = 50)	*p*‐value
Age (years)	46.46 ± 15.59	44.73 ± 16.65	45.70 ± 15.92	0.706
Age group (years)				0.952
< 20	2 (7.1)	2 (9.1)	4 (8.0)	
20–30	3 (10.7)	3 (13.6)	6 (12.0)	
31–40	3 (10.7)	3 (13.6)	6 (12.0)	
> 40	20 (71.4)	14 (63.6)	34 (68.0)	
Educational level				0.266
Primary	5 (17.9)	3 (13.6)	8 (16.0)	
Senior high school	6 (21.4)	7 (31.8)	13 (26.0)	
University	13 (46.4)	12 (54.5)	25 (50.0)	
Postgraduate	4 (14.3)	0 (0)	4 (8.0)	
Marital status				0.983
Single	7 (25.0)	6 (27.3)	13 (26.0)	
Married	17 (60.7)	13 (59.1)	30 (60.0)	
Divorced	4 (14.3)	3 (13.6)	7 (14.0)	
BMI grade				**0.002**
Underweight	2 (7.1)	2 (9.1)	4 (8.0)	
Normal	3 (10.7)	12 (54.5)	15 (30.0)	
Overweight	12 (42.9)	7 (31.8)	19 (38.0)	
Obese	11 (39.3)	1 (4.5)	12 (24.0)	
Weight (kg)	71.04 ± 16.02	66.55 ± 12.98	69.06 ± 14.79	0.291
BMI (kg/m^2^)	28.55 ± 6.50	23.61 ± 4.85	26.38 ± 6.29	**0.005**
FBG (mmol/L)	5.40 ± 0.47	5.1 ± 0.65	5.31 ± 0.56	0.193
Height (cm)	159.5 (150–162)	169.5 (162.25–172)	162 (150–169.25)	**< 0.001**
HbA1C (%)	5.15 (4.9–5.58)	5.1 (4.9–5.60)	5.10 (4.90–5.60)	0.868
eAG	5.62 (5.3–6.16)	5.49 (5.22–6.3)	5.55 (5.26–6.21)	0.814
2‐h post‐glucose‐load glucose (mmol/L)	8.85 (6.33–10.46)	7.2 (6.45–8.63)	7.65 (6.38–9.83)	0.135

*Note:* Bold *p* values are indicate statistical significance.

Abbreviation: eAG, estimated average glucose.

Figure 1 shows the proportions of participants meeting the diabetes thresholds. Two participants (4.0%) met the HbA1c criterion, seven (14.0%) met the 2‐h post‐glucose‐load criterion, and none met the FBG criterion. These estimates are based on a small number of positive cases and should therefore be interpreted cautiously.

**Figure 1:**
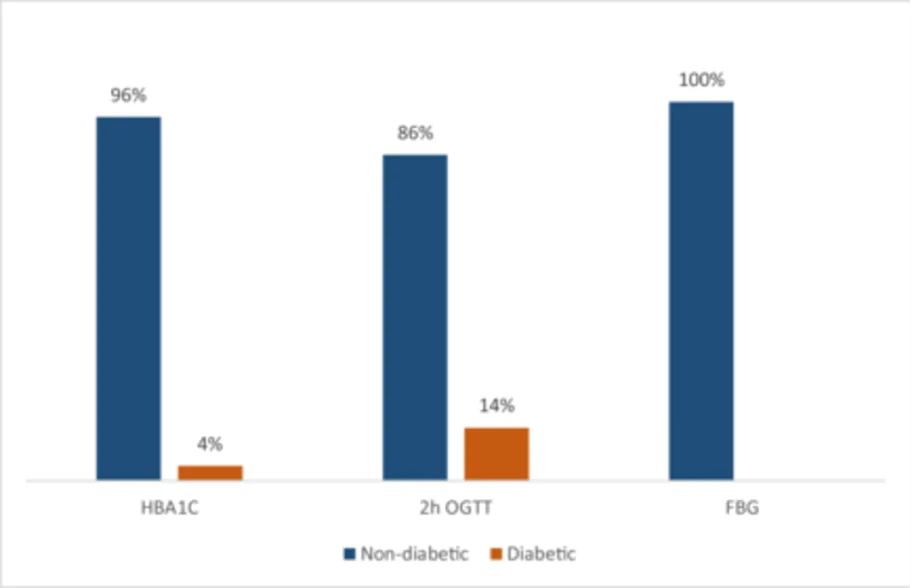
Proportion of participants meeting diabetes thresholds according to HbA1c, 2‐h post‐glucose‐load glucose and FBG. FBG, fasting blood glucose; HbA1c, glycated hemoglobin.

Table 2 presents the sociodemographic characteristics and BMI of participants in relation to diabetes status. No significant association was observed between diabetes and age (OGTT: *p* = 0.564; HbA1c: *p* = 0.388); most participants meeting the 2‐h post‐glucose‐load criterion for diabetes mellitus (85.7%) were above 40 years. Sex distribution showed no significant difference (OGTT: *p* = 0.322; HbA1c: *p* = 0.691), with more females (71.4%) classified as positive by the post‐load criterion. Educational level was significantly associated with diabetes classification using the post‐load criterion (*p* = 0.013), but not HbA1c (*p* = 0.555), as diabetes prevalence was higher among those with only elementary education. However, this finding should be interpreted cautiously because it was based on only seven participants meeting the post‐load criterion for diabetes. No comparable association was observed using HbA1c criteria. Marital status showed borderline significance by HbA1c (*p* = 0.052) but not OGTT (*p* = 0.437); all HbA1c‐defined diabetics were single. BMI was not significantly associated with diabetes (HbA1c: *p* = 0.796; OGTT: *p* = 0.881); among participants meeting the post‐load criterion for diabetes, 28.6% each were obese, overweight, or normal, and 14.3% were underweight.

**Table 2:** Sociodemographic characteristics and BMI of study participants in relation to diabetes status of participants.

	HBA1C			The 2‐h post‐glucose‐load classification		
Variables	Non‐diabetic (*N* = 48)	Diabetic (*N* = 2)	*p*‐value	Non‐diabetic (*N* = 43)	Diabetic (*N* = 7)	*p*‐value
Age (years)			0.388			0.564
< 20	4 (8.3)	0 (0)		4 (9.3)	0 (0)	
20–30	6 (12.5)	0 (0)		6 (14.0)	0 (0)	
31–40	5 (10.4)	1 (50.0)		5 (11.6)	1 (14.3)	
> 40	33 (68.8)	1 (50.0)		28 (65.1)	6 (85.7)	
Sex			0.691			0.322
Female	27 (56.3)	1 (50.0)		23 (53.5)	5 (71.4)	
Male	21 (43.8)	1 (50.0)		20 (46.5)	2 (28.6)	
Educational level			0.555			**0.013**
Primary	8 (16.7)	0 (0)		5 (11.6)	3 (42.9)	
Senior High	13 (27.1)	0 (0)		13 (30.2)	0(0)	
University	23 (47.9)	2 (100)		23 (53.5)	2 (28.6)	
Postgraduate	4 (8.3)	0 (0)		2 (4.7)	2 (28.6)	
Marital status			0.052			0.437
Single	11 (22.9)	2 (100)		11 (25.6)	2 (28.6)	
Married	30 (62.5)	0 (0)		27 (62.8)	3 (42.9)	
Divorced	7 (14.6)	0 (0)		5 (11.6)	2 (28.6)	
BMI			0.796			0.881
Underweight	4 (8.3)	0 (0)		3 (7.0)	1 (14.3)	
Normal	14 (29.2)	1 (50.0)		13 (30.2)	2 (28.6)	
Overweight	18 (37.5)	1 (50.0)		17 (39.5)	2 (28.6)	
Obese	12 (25.0)	(0)		10 (23.3)	2 (28.6)	

*Note:* Bold *p* values are indicate statistical significance.

Table 3 presents lifestyle and dietary practices according to diabetes status. No statistically significant associations were identified between diabetes status and exercise, eating late at night, dietary restrictions, alcohol consumption, or reported medical conditions using either HbA1c or OGTT criteria (all *p* > 0.05). Although a greater proportion of participants diagnosed with diabetes mellitus by OGTT reported not engaging in regular physical activity (85.7%), this observation did not reach statistical significance.

**Table 3:** Lifestyle and dietary practices of study participants by diabetes status.

	HbA1C			The 2‐h post‐glucose‐load classification		
Variables	Non‐diabetic (*N* = 48)	Diabetic (*N* = 2)	*p*‐value	Non‐diabetic (*N* = 43)	Diabetic (*N* = 7)	*p*‐value
Exercise			0.735			0.062
Yes	23 (47.9)	1 (50.0)		23 (53.5)	1 (14.3)	
No	25 (52.1)	1 (50.0)		20 (46.5)	6 (85.7)	
Eat late at night			0.486			0.357
Yes	13 (27.1)	1 (50.0)		13 (30.2)	1 (14.3)	
No	35 (72.9)	1 (50.0)		30 (69.8)	6 (85.7)	
Dietary restrictions			0.737			0.677
Yes	7 (14.6)	0 (0)		6 (14.0)	1 (14.3)	
No	41 (85.4)	2 (100)		37 (86.0)	6 (85.7)	
Take alcohol			0.192			0.454
Yes	4 (8.3)	1 (50.0)		5 (11.6)	0 (0)	
No	44 (91.7)	1 (50.0)		38 (88.4)	7 (100)	
Any medical conditions			0.808			0.454
Yes	5 (10.4)	0 (0)		5 (11.6)	0 (0)	
No	43 (89.6)	2 (100)		38 (88.4)	7 (100)	

Table 4 presents the clinical characteristics of participants by diabetes status. No significant association was found between classic diabetes symptoms and diabetes status using either HbA1c or post‐load criteria (*p* > 0.05). Among HbA1c‐defined diabetics, 50% reported excessive thirst, 100% frequent urination, and 50% blurred vision, with none reporting slow‐healing wounds or weight loss. Similarly, participants meeting the post‐load criterion for diabetes mellitus reported excessive thirst (3 cases), frequent urination (4), blurred vision (3), numbness or tingling (2), and no cases of slow‐healing wounds or unexplained weight loss. Because only two participants were classified as diabetic by HbA1c and seven by the post‐load criterion, these findings should be interpreted with caution.

**Table 4:** Clinical characteristics stratified by diabetes status of participants.

	HbA1C			The 2‐h post‐glucose‐load classification		
Variables	Non‐diabetic (*N* = 48)	Diabetic (*N* = 2)	*p*‐value	Non‐diabetic (*N* = 43)	Diabetic (*N* = 7)	*p*‐value
Excessive thirst			0.569			0.446
Yes	16 (33.3)	1 (50.0)		14 (32.6)	3 (42.9)	
No	32 (66.7)	1 (50.0)		29 (67.4)	4 (57.1)	
Frequent urination			0.189			0.362
Yes	20 (41.7)	2 (100)		18 (41.9)	4 (57.1)	
No	28 (58.3)	0 (0)		25 (58.1)	3 (42.9)	
Unexplained weight loss			0.845			0.536
Yes	4 (8.3)	0 (0)		4 (9.3)	0 (0)	
No	44 (91.7)	2 (100)		39 (90.7)	7 (100)	
Blurry vision			0.755			0.500
Yes	24 (50.0)	1 (50.0)		22 (51.2)	3 (42.9)	
No	24 (50.0)	1 (50.0)		21 (48.8)	4 (57.1)	
Slow‐healing sores or cuts			0.808			0.454
Yes	5 (10.4)	0 (0)		5 (11.6)	0 (0)	
No	43 (89.6)	2 (100)		38 (88.4)	7 (100)	
Numbness or tingling			0.514			0.643
Yes	14 (29.2)	0 (0)		12 (27.9)	2 (28.6)	
No	34 (70.8)	2 (100)		31 (72.1)	5 (71.4)	

Table 5 presents the diagnostic agreement between HbA1c and the 2‐h post‐glucose‐load classification. The overall observed agreement was 86.0%; however, Cohen's kappa coefficient indicated only slight agreement beyond chance (*κ* = 0.17, *p* = 0.134). The apparent discrepancy between the high crude agreement and the low kappa value is likely attributable to the low prevalence of diabetes in the study population, whereby agreement is driven predominantly by the large number of participants classified as non‐diabetic by both methods. The underlying 2 × 2 contingency tables for HbA1c and FBG relative to the reference classification are provided in File S1.

**Table 5:** Diagnostic Agreement between HbA1c and 2‐h post‐glucose‐load classification in Diagnosing Diabetes Mellitus.

	HbA1c				
	Non‐diabetic (*n*)	Diabetic (*n*)	kappa	*p*‐value	Agreement
2‐h post‐glucose‐load classification			0.17	0.134	86%
Non‐diabetic	42	1			
Diabetic	6	1			

Table 6 presents the diagnostic performance of HbA1c and FBG using 2‐h post‐glucose‐load classification as the reference standard. HbA1c demonstrated a sensitivity of 14.3% (95% CI: 0.4%–57.9%), specificity of 97.9% (95% CI: 87.7%–99.9%), PPV of 50.0% (95% CI: 1.3%–98.7%), and NPV of 87.5% (95% CI: 74.7%–95.3%). In contrast, FBG demonstrated 0.0% sensitivity (95% CI: 0.0%–41.0%) and 100.0% specificity (95% CI: 91.8%–100.0%), indicating that it failed to identify any participants meeting the post‐load criterion for diabetes. The NPV of FBG was 86.0% (95% CI: 73.3%–94.2%). These estimates should be interpreted with caution because they were derived from only seven participants meeting the post‐load criterion for diabetes, resulting in wide confidence intervals, particularly for sensitivity and PPV.

**Table 6:** Diagnostic Performance of HbA1c Compared with the 2‐h post‐glucose‐load reference classification.

Diagnostic test	Sensitivity % (95% CI)	Specificity % (95% CI)	Positive predictive value % (95% CI)	Negative predictive value % (95% CI)
HbA1c	14.3 (0.4–57.9)	97.9 (87.7–99.9)	50.0 (1.3–98.7)	87.5 (74.7–95.3)
Fasting blood glucose (FBG)	0.0 (0.0–41.0)	100.0 (91.8–100.0)	Not estimable	86.0 (73.3–94.2)

## 4 Discussion

This study compared the performance of FBG and HbA1c with OGTT for diagnosing diabetes mellitus among adults at increased risk of diabetes mellitus attending a tertiary healthcare facility in Ghana. The principal findings were that the 2‐h post‐glucose‐load criterion identified the highest proportion of diabetes cases, whereas HbA1c detected only a minority of participants meeting the post‐load criterion, and FBG failed to identify any participant with diabetes. Although both HbA1c and FBG demonstrated high observed specificity, their low sensitivity estimates were based on only seven by the post‐load criterion, participants and should therefore be interpreted cautiously.

The higher proportion of diabetes mellitus detected using the post‐load criterion may reflect the occurrence of post‐load hyperglycemia before fasting hyperglycemia during the progression of type 2 diabetes mellitus. Consequently, individuals with impaired glucose regulation may demonstrate normal fasting glucose concentrations while exhibiting elevated post‐load glucose levels detectable by OGTT. This provides a possible explanation for the observed differences between tests, although the present study cannot establish the underlying mechanism. OGTT remains an established method for assessing glucose tolerance [9], but these findings alone do not establish its superiority in this population.

HbA1c demonstrated low sensitivity (14.3%) but high specificity (97.7%) relative to the post‐load reference classification. This indicates that while HbA1c correctly identified 42 of the 43 reference‐negative participants, it missed the majority of participants meeting the post‐load criterion for diabetes mellitus. Of the two positive HbA1c results, one was also positive by the post‐load criterion, while 42 of the 48 negative HbA1c results were negative by post‐load criterion. These findings align with studies which also reported lower sensitivity but high specificity of HbA1c compared to OGTT [5, 12, 13]. The low sensitivity suggests that HbA1c identified fewer diabetes mellitus cases than post‐load criterion in this sample, especially among individuals with isolated postprandial hyperglycemia or early glucose intolerance that OGTT captures. However, the small number of reference‐positive participants limits the precision of the sensitivity estimate rather than necessarily explaining its low value. Similarly, the high observed specificity does not establish HbA1c's reliability for confirming diabetes, particularly because only two participants had positive HbA1c results. In addition, HbA1c measurements may be influenced by biological factors independent of glycemia, including anemia, hemoglobinopathies, and altered erythrocyte turnover, which are relevant considerations in sub‐Saharan African populations. Other studies have reported different patterns of diagnostic performance or diabetes classification using HbA1c and OGTT [7, 14, 15]. The variations in the findings may reflect differences in study populations and measurement methods, while differences in sample size affect the precision of the estimates.

The study observed no significant association between diabetes mellitus status defined by HbA1c or the post‐load criterion and age, sex, or BMI except for educational level which was significantly associated with diabetes mellitus by defined by the post‐load criterion. However, this association should be interpreted cautiously because only seven participants were OGTT‐positive and several educational categories contained few participants. Similarly, study conducted in Sweden by [16] showed no significant association between diabetes mellitus status by HbA1c or OGTT and age, sex, or BMI. In contrasts, with some literature noting age and ethnicity‐related variations affecting HbA1c accuracy carried out at Emory University in Atlanta, USA who found HbA1c rises with age, potentially reducing specificity in older adults [17]. The insignificant sex and BMI associations are consistent with findings by Janghorbani and others in Iran, though sex differences in glucose metabolism patterns may influence test performance [18]. The variations in the findings may be due the sample size used and study population.

The present study observed no significant association between diabetes mellitus status defined by HbA1c or post‐load criterion and variables such as exercise, eating late at night, dietary restrictions, alcohol intake, and any medical conditions. This aligns with a study that observed no significant association between alcohol intake and HbA1c levels [19]. Another study also found out that eating late meals was not associated with abnormal glucose metabolism [20]. On the other hand, Hu et al. observed an inverse association between physical activity and type 2 diabetes mellitus [21]. These comparisons remain exploratory because the small number of positive cases and sparse cell counts limited the ability to detect associations.

The study showed 86% diagnostic agreement between HbA1c and the post‐load reference classification but Cohen's kappa indicated only slight agreement (*κ* = 0.17), suggesting limited concordance beyond chance which align with this study in Athens, Greece [22]. This apparent discrepancy reflects the influence of disease prevalence and the marginal distributions of the tests on the kappa statistic. Because most participants were classified as non‐diabetic by both methods, overall agreement was largely driven by concordant negative results rather than concordant positive diagnoses. Specifically, 42 of the 43 concordant classifications were negative. Percentage agreement alone therefore does not adequately describe agreement on positive cases. However, kappa must also be interpreted cautiously: with only two HbA1c‐positive and seven reference‐positive participants, changes in the classification of a few individuals could substantially alter the estimate.

An important finding of this study was that FBG failed to identify any participant meeting the post‐load criterion for diabetes mellitus. Although this may reflect abnormalities confined to post‐load glucose metabolism, the observed sensitivity of 0% was based on only seven reference‐positive participants and had a wide 95% confidence interval of 0.0%–41.0%. FBG also showed 100% specificity, but this arose because all participants were classified as negative; its PPV could not be estimated. Although FBG remains a convenient and inexpensive diagnostic tool, these findings are insufficient to conclude that it has limited diagnostic utility in the wider population. A larger study could produce different estimates of its sensitivity and specificity.

This study has several limitations. First, the small sample size reduced statistical power and resulted in wide confidence intervals around the estimable diagnostic performance measures, thereby limiting the precision of sensitivity, specificity, PPV, and NPV. Only seven participants were positive by the post‐load criterion and two by HbA1c; consequently, the sensitivity and PPV estimates depended on very small numbers. A change in classification for one reference‐positive participant would alter sensitivity by approximately 14 percentage points. These sparse counts also limit the stability of kappa and the strength of conclusions about comparative diagnostic performance. Second, participants were recruited using purposive sampling from a single tertiary healthcare facility, which may have introduced spectrum bias and limits the generalisability of the findings to the wider Ghanaian population. Third, the hospital‐based study population consisted exclusively of adults at increased risk of diabetes and therefore may not reflect the diagnostic performance of these tests in community‐based or lower‐risk populations. Subgroup analyses were also limited by sparse cell counts and multiple comparisons and should be considered exploratory. Finally, the cross‐sectional design precludes assessment of temporal changes in glycaemic status or progression to diabetes.

Despite these limitations, this study provides important preliminary evidence regarding the comparative performance of FBG, HbA1c, and OGTT among adults at increased risk of diabetes in Ghana. To our knowledge, few studies have directly compared these diagnostic approaches within this population using the 2‐h post‐glucose‐load classification as the reference standard. The findings contribute locally relevant evidence that may inform future diagnostic strategies and larger multicentre studies. Future studies should include larger, multicentre populations to improve the precision and generalisability of diagnostic performance estimates. Further research should also evaluate combined diagnostic algorithms incorporating FBG, HbA1c, and OGTT to determine whether such approaches improve diabetes detection among high‐risk individuals. However, the potential benefit of combined testing remains a hypothesis requiring formal evaluation rather than a conclusion supported by the present study.

## 5 Conclusion

In this exploratory hospital‐based study, the 2‐h post‐glucose‐load criterion identified more participants with diabetes‐range results than HbA1c or FBG. Agreement between HbA1c and the post‐load classification was predominantly attributable to concordant negative results. The small sample and limited number of positive cases resulted in substantial uncertainty and prevent firm conclusions about comparative diagnostic performance. Larger studies using standardised measurement procedures and sufficient numbers of positive cases are needed before these findings can inform diagnostic practice.

## Author Contributions

**Emmanuel Okrah:** resources, writing – original draft, formal analysis, data curation, funding acquisition. **Dawudu Abdullah:** writing – original draft, resources, formal analysis, data curation, funding acquisition. **Beatrice Manu:** writing – original draft, resources, data curation, formal analysis, funding acquisition. **James Kojo Prah:** supervision, project administration. **Samuel Amoah:** supervision, writing – review and editing. **Benjamin Nyane:** supervision, project administration. **Gloria N. Nartey:** writing – review and editing, project administration. **Eugenia N. A. Ayetey:** writing – review and editing, formal analysis. **Ibrahim Anyass Goumboundi:** supervision, project administration, methodology, writing – review and editing, writing – original draft, visualization, investigation. **Justice Afrifa:** methodology, writing – review and editing, supervision, project administration, conceptualization, software. **Richard K. D. Ephraim:** supervision, conceptualization, methodology, validation, writing – review and editing, project administration.

## Funding

The authors have nothing to report.

## Ethics Statement

Ethical approval was obtained from the Institutional Review Board of the University of Cape Coast Hospital (UCC‐184/2025). All data were kept confidential and used solely for research purposes. Written informed consent was obtained from all participants. The study was conducted in accordance with the ethical principles outlined in the Declaration of Helsinki.

## Consent

The authors have nothing to report.

## Conflicts of Interest

The authors declare no conflicts of interest.

## AI Use Disclosure

No AI tool was used in the preparation of this manuscript.

## Transparency Statement

The authors affirm that this manuscript is an honest, accurate, and transparent account of the study being reported; that no important aspects of the study have been omitted; and that any discrepancies from the study as originally planned have been explained. All authors have read and approved the final version of the manuscript. Ibrahim Anyass Goumboundi had full access to all of the data in this study and takes complete responsibility for the integrity of the data and the accuracy of the data analysis.

## Supporting information


Supporting File

## Data Availability

The data that support the findings of this study are available from the corresponding author upon reasonable request.
