# Supplementary material for: Diagnostic Utility of Fasting Blood Glucose and Glycated Hemoglobin Compared to Oral Glucose Tolerance Test for Diabetes Mellitus: A Cross‐Sectional Study
Source: Health Sci Rep. 2026 Nov 10;9(11):e73507. doi: 10.1002/hsr2.73507 (PMC13749272; doi:10.1002/hsr2.73507)
Supplement: Supplementary file 1 [file HSR2-9-e73507-s001.docx]

# **Table S1. HbA1c versus the post-load reference classification**

| **HbA1c classification** | **Reference positive** | **Reference negative** | **Total** |
| --- | --- | --- | --- |
| **Positive** | 1 | 1 | 2 |
| **Negative** | 6 | 42 | 48 |
| **Total** | 7 | 43 | 50 |

**Table S2. FBG versus the post-load reference classification**

| **FBG classification** | **Reference positive** | **Reference negative** | **Total** |
| --- | --- | --- | --- |
| Positive | 0 | 0 | 0 |
| Negative | 7 | 43 | 50 |
| Total | 7 | 43 | 50 |
